# Addressing Obesity in Preconception, Pregnancy, and Postpartum: A Review of the Literature

**DOI:** 10.1007/s13679-022-00485-x

**Published:** 2022-11-01

**Authors:** Siew Lim, Cheryce Harrison, Emily Callander, Ruth Walker, Helena Teede, Lisa Moran

**Affiliations:** 1 Monash Centre for Health Research and Implementation, Monash University, Clayton, VIC Australia; 2 Health Systems and Equity, Eastern Health Clinical School, Monash University, Boxhill, VIC Australia

**Keywords:** Preconception, Pregnancy, Postpartum, Weight, Lifestyle

## Abstract

***Purpose of Review*:**

Reproductive-aged women (aged 19 to 50 years) are a key population warranting focused research for the prevention of overweight and obesity. This review highlights the importance of addressing weight before, during and after pregnancy.

***Recent Findings*:**

Obesity decreases fertility during the preconception period; increases the risk of adverse pregnancy outcomes including gestational diabetes, pre-eclampsia and caesarean section and postpartum weight retention; and increases the long-term health risks for both the mother and offspring. Despite overwhelming efficacy evidence on solutions, there are significant implementation gaps in translating this evidence into pragmatic models of care and real-world solutions. Interventions during preconception, pregnancy and postpartum are likely to be cost-effective or cost-saving, with future investigation needed in the preconception and postpartum period.

***Summary*:**

International clinical guidelines and public health policies are needed for a concerted effort to prevent unhealthy weight gain in these life stages and to reverse the significant adverse health outcomes for women and the next generation.

## Introduction

The global prevalence of obesity has increased over the last four decades, from 3 to 11% in men and from 6 to 14% in women [1]. If these trends continue, it is expected that the global prevalence of obesity would reach 18% in men and 21% in women by 2025 [1]. Reproductive-aged women gain weight more rapidly than men and women of other age groups [2, 3] with longitudinal studies reporting more weight gain over 10 years (6.3 kg) for women aged 18–50 years [4] compared to women aged 50 and over [5]. Around 40% of women aged > 18 years have an overweight BMI and 15% have obesity [6]. Pregnancy is a critical window in the reproductive life course that drives weight gain and maternal adiposity [7, 8]. Approximately 40% of women commence pregnancy overweight or obese [9] and the majority gain excess weight in pregnancy according to BMI specific Institute of Medicine (IOM) recommendations (now, the National Academy of Medicine [10, 11]). Our previous meta-analysis incorporating over 1.3 million pregnancies from 23 studies across 10 countries highlighted that only 30% of women gained within gestational weight gain (GWG) guidelines, with 47% of women exceeding recommendations and the remaining 23% gaining below guidelines [12]. Excessive GWG could lead to postpartum weight retention, which is also a major contributing factor to obesity in women [13]. Women retain between 0.5 and 3 kg postpartum, and 25–47% of women retain ≥4.5 kg by 1 year postpartum [14–18].

The World Health Organization (WHO) recognises suboptimal diet and low physical activity levels as key contributors to obesity [19]. This is mainly driven by changes in the global food system and the production of more affordable, processed food that is aggressively marketed toward consumers [20]. Within populations, the interaction between environment and individual factors results in the problem of obesity being disproportionately greater among people with socio-economic disadvantage. Obesity rates are inversely associated with socioeconomic status, especially in women in developed countries [21]. However, the reverse could be true in low-income countries [22]. This is related to the recognised influence of key sociodemographic factors such as ethnicity, marital status, employment, education, income, and geographic location on diet and physical activity in women [23]. Unlike other major public health crises such as infectious diseases or tobacco use, there are currently no successful examples of weight reduction at a population level due to public health measures [20].

Obesity has significant implications for preconception, pregnancy, and postpartum periods. While the definition of pregnancy is unequivocal, the definition of preconception and postpartum warrants further description. In the Lancet series on preconception, preconception was defined variously as the time right before conception, the time reflecting the intentionality of a couple to conceive or the time to address preconception risk factors such as diet and obesity [24]. According to the latter definition, preconception includes all women of reproductive age to be targeted in public health initiatives. There is no consensus on the definition of the postpartum period. From a chronic disease prevention perspective, evidence on diabetes prevention in women who had high-risk pregnancies (e.g. gestational diabetes) suggests that the appropriate period for intervention could be anytime between a child’s birth and 5 years postpartum [25]. In this review, we have defined postpartum as the time from birth to 2 years, to reflect the importance of the offspring’s first 1000 days [26]. Recent reviews on weight in preconception, pregnancy and postpartum addressed the issue of weight stigma and provided an overview of clinical practice guidelines in these life stages [27, 28]. However, these reviews do not comprehensively cover the impact of obesity on these life stages nor the implementation issues and gaps in addressing obesity in these life stages. 

Therefore, this review aims to provide an overview of the impact of obesity in women across preconception, pregnancy and postpartum periods and the issues around addressing weight and lifestyle during these life stages. We will achieve this overarching goal by describing the significance of weight and lifestyle management at these reproductive life stages, implementation challenges of weight and lifestyle management and current evidence on effective solutions targeting individuals at these life stages and gaps in current knowledge. While we acknowledge the importance of prevention at a population level, this review will focus on evidence targeting individuals. We have searched the Medline/PubMed databases for these keywords to provide a comprehensive review to this goal: preconception, women, pregnancy, health, overweight, obesity, postpartum, lifestyle, weight, gestational weight gain, antenatal lifestyle intervention, implementation, cost-effectiveness and economic evaluation.

## Preconception

### Significance of Weight and Lifestyle in the Preconception

Overweight and obesity during the preconception period can have significant health impacts on women and their offspring. Overweight and obesity are associated with infertility and can influence how infertility is treated [29, 30]. For those who do conceive, maternal overweight and obesity increase the risk of excessive GWG [9], subsequent postpartum weight retention and lifelong obesity [31]. A higher preconception BMI independently increases pregnancy complications including gestational diabetes, preeclampsia, caesarean section and large-for-gestational-age (LGA) infants [32]. Offspring born to mothers who are overweight or who have obesity in early pregnancy are more likely to become overweight or develop obesity themselves [33, 34]. Epigenetic programming that occurs at conception and throughout pregnancy predisposes children born to mothers with obesity to a range of chronic metabolic conditions including type 2 diabetes and heart disease [35, 36]. Maternal overweight or obesity originates long before pregnancy and achieving an optimal weight for conception may take many months or even years for some women to achieve. Therefore, public health interventions regarding aspects of lifestyle linked to maternal obesity, such as physical activity and nutrition, should occur much earlier in a woman’s life [37].

### Challenges to Lifestyle Management During Preconception

Knowledge and awareness of optimal diet and physical activity levels in the preconception period have been identified as both barriers and enablers of optimising these lifestyle behaviours [38]. Developing public health interventions that promote awareness of optimal preconception lifestyle behaviours is challenging because the target audience is so diverse. In 2005, the US National Preconception Health Consumer Workgroup developed a social marketing plan to increase women’s awareness of preconception health and promote healthy lifestyle behaviours [39]. Two market segments were identified, pregnancy ‘intenders’ (planning to become pregnant in the next year) and pregnancy ‘non-intenders’ (not planning a pregnancy but when lifestyle behaviours can impact future pregnancies) [39, 40]. Pregnancy ‘intenders’ are generally motivated by a desire to become pregnant and resonate with the concept of preconception health, including preconception and pregnancy terminology in health promotion messages. On the other hand, pregnancy ‘non-intenders’ are motivated by improving their health and wellbeing and tend not to resonate with the concept of improving their preconception health or terminology related to preconception and pregnancy in health promotion messages. Therefore, public health interventions that promote awareness of optimal preconception lifestyle behaviours targeted at pregnancy ‘non-intenders’ are unlikely to resonate with women if they are explicitly preconception focused [39].

Public awareness of the importance of preconception care is low, even in pregnancy intenders [41, 42], indicating that public health interventions relating to preconception care are yet to engage women or promote health behaviours. For example, in a highly advantaged sample of women (*n* = 294) living in Australia, 90% were unaware of reproductive life planning [43]. Interestingly, when comparing intenders (*n* = 121) and non-intenders (*n* = 173), there was no significant difference in the uptake of medical screening, routine health checks and immunisations and weight gain in the past 12 months (54.4% had gained weight). On the other hand, a higher proportion of pregnancy intenders were taking a prenatal supplement (intenders 44.6%, non-intenders 17.9%, *p* < 0.001) [42], suggesting public health messages around this aspect of preconception health have made some headway and that well-considered public health messages around other aspects of preconception health behaviours and the importance of engaging with preconception care may be of benefit. Barriers to the provision of preconception care encountered by health professionals, including short consultation times, lack of guidelines and inadequate remuneration [44, 45] also need to be addressed [46, 47].

### Intervention Studies Addressing Lifestyle Preconceptions

Lifestyle interventions for obesity prevention targeted to pregnancy ‘intenders’ are of benefit. For example, anovulatory women with obesity (*n* = 67) enrolled in a prospective six-month weight-loss trial lost 10.2kg (±4.3kg), 90% resumed ovulation within this timeframe, and 67% achieved a live birth, compared with none in the comparator group (*n* = 20). Women in the intervention group also achieved improvements in measures of self-esteem, anxiety and depression [48]. A systematic review of 21 randomised controlled trials (RCTs) assessed non-pharmacological interventions for women diagnosed with infertility and with a BMI > 25 mg/m^2^ [49]. Meta-analyses of combined diet and physical activity interventions (*n* = 4) found that the pregnancy rate was higher for intervention, compared with control (RR 1.63; 95%CI, 1.21–2.20; *p* = 0.01) and the live birth rate was higher for intervention, compared with control (RR 1.57; 95%CI, 1.11–2.22; *p* = 0.01). Results were less conclusive when other non-pharmacological interventions such as diet only and behavioural modification counselling were added to the meta-analyses [49]. For women who are overweight or with obesity, lifestyle interventions based on diet and physical activity leading to weight loss before conception can increase fertility and live birth rates. While prospective RCTs are required to assess the longer-term benefits of preconception lifestyle and weight-loss interventions [36], this should not stop health providers from supporting women to achieve a healthy weight during the preconception period.

### Gaps

Few interventions have explored whether public health lifestyle interventions that are explicitly preconception focused on ‘non-intenders’ can improve preconception health or weight status. Ongoing work in the USA is testing a virtual patient advocate called ‘Gabby’ that supports African American women to decrease preconception health risks under 13 different categories including substance use, exposure to environmental toxins, diet and physical activity, to name a few [50, 51]. In a state-based [50] and national [52] RCTs, Gabby was found to support African American women, both ‘intenders’ and ‘non-intenders’, to decrease their preconception health risks over time. The degree to which Gabby supported women to make healthy diet and physical activity changes or achieve an optimal preconception weight has not been specifically measured; however, this research provides evidence that pregnancy ‘non-intenders’ may be reached via digital health-based interventions.

The ‘Show Your Love’ Campaign is another example of a preconception health intervention targeted at pregnancy ‘non-intenders’ [39]. Launched in 2013 by the US Centres of Disease Control, ‘Show Your Love’ used a social marketing framework to develop a campaign that would resonate with both pregnancy ‘intenders’ and ‘non-intenders’ The campaign for ‘non-intenders did not use preconception terminology but instead marketed the achievement of personal goals, empowerment and health and wellbeing [39]. To the authors’ knowledge, this campaign has not been formally evaluated. ‘Show Your Love’ highlights the importance of distinguishing between market segments within a broad population group and targeting interventions to meet the needs of the target audience.

Community awareness of the importance of a healthy weight and preconception health for pregnancy is low [39, 53]. Many young people graduate from school with very little understanding of their preconception health [54, 55]. Opportunities for formal sexual and reproductive health education, including achieving and maintaining an optimal preconception weight, are limited after secondary school so sexual and reproductive education during a young person’s time at primary and secondary school may be the only time they receive any information related to this important topic area [56]. This is a significant problem, and further work should be done within schools to increase community awareness of the importance of preconception health.

## Pregnancy

### Significance of Weight and Lifestyle in Pregnancy

Inadequate GWG, below or in excess of National Academy of Medicine guidelines, is common, occurring in 23% and 47% of pregnancies, respectively [9]. Compared to GWG within recommendations, GWG below guidelines is associated with a higher risk of small-for-gestational-age birth (OR 1.53 [95% CI, 1.44–1.64) and preterm birth (OR 1.70 [95% CI: 1.32–2.20]) and lower risk of large-for-gestational-age birth (OR, 0.59 [95% CI: 0.55–0.64]) and macrosomia (OR 0.60 [95% CI: 0.52–0.68]). GWG in excess of guidelines is associated with a higher risk of LGA birth (odds ratio [OR] 1.85, [95% CI: 1.76–1.95]), macrosomia (OR 1.95 [95% CI: 1.79–2.11]) and caesarean delivery (OR 1.30 [95% CI: 1.25–1.35). Epigenetic impacts are significant, with children being three times more likely to develop obesity when GWG exceeds guidelines, independent of maternal BMI [57]. Excessive GWG also contributes to obesity in women [58], thereby increasing the risk of non-communicable disease in women in the long term. Pre-existing obesity exacerbates the risk with children born to mothers with obesity twice as likely to develop childhood obesity, independent of maternal age, race, parity, education, gestational weight gain, child gender and birth weight of the child [59].

### Challenges to Lifestyle Management During Pregnancy

Women commonly experience significant and unique challenges to maintaining healthy lifestyle behaviours during pregnancy for reasons including fatigue, intermittent or persistent nausea or the more severe *hyperemesis gravidarum* (i.e. severe nausea and vomiting occurring in 1–2% of pregnancies) time limitations and ongoing work and/or child care responsibilities [60, 61]. Despite pregnancy being considered a ‘teachable moment’ for obesity prevention by some health professionals and researchers [62], in reality, lifestyle change related to weight management is complex with socio-ecological considerations including the environment in which women live and the influence of social networks, community, healthcare and government on individual health. However, evidence suggests that behaviour change during pregnancy can be optimised with adequate support [63, 64].

### Intervention Studies Addressing Lifestyle During Pregnancy

Pregnancy is recognized as a critical window to optimize maternal health behaviours and lifestyle to benefit the future health of both mother and child [65]. Consequently, a concentrated research effort has been placed on antenatal lifestyle interventions for improving GWG outcomes [63, 64]. A recent US Prevention task force systematic review and meta-analyses of 68 behavioural lifestyle intervention trials reported a favourable reduction in GWG (mean difference −1.02 kg; 95% confidence interval (CI): −1.30 to −0.75; 55 studies; *n*=20,090) with an associated reduction in GDM (relative risk [RR], 0.87 [95% CI, 0.79 to 0.95, 43 trials, *n* = 19,752) and emergency caesarean delivery (RR, 0.85 [95% CI, 0.74 to 0.96]; 14 trials, *n* = 7,520) risk [63]. These findings are supported by our meta-analysis of 117 lifestyle interventions incorporating structured diet and/or exercise or unstructured mixed lifestyle interventions (with or without behavioural counselling) involving 34,546 women [66]. Overall, lifestyle intervention reduced GWG (mean difference −1.15 kg; 95%CI: −1.40 to −0.91; 99 studies; 29,247 women), gestational diabetes (odds ratio (OR) 0.79; 95% CI: 0.70 to 0.89; 67 studies; 24,371 women) and total adverse maternal outcomes (OR 0.89; 95% CI: 0.84 to 0.94; I2=28%) [66]. With Level 1 evidence demonstrating the efficacy of lifestyle interventions alongside cost-effectiveness and potential cost savings [67], there is now a strong mandate for the translation and implementation of effective interventions into policy and practice [63, 68]. Strategies to generate knowledge and public health impact for the prevention of weight gain in this critical window offers potential for major benefits to reproductive, cardio-metabolic and psychological health in women of reproductive age and their families.

### Gaps

Despite extensive research demonstrating efficacy and cost-effectiveness [66, 69] of lifestyle intervention in pregnancy, significant challenges remain preventing the translation of evidence into real-world implementation. To date, no country has implemented systems practice- and policy-level evidence-based strategies targeting preconception and pregnancy life stages to prevent excess weight gain and GWG during antenatal care and obesity development more broadly. Key challenges include a need to disentangle the complexity of lifestyle interventions to enhance specificity in understanding exactly what should be implemented, how and by whom, supported by rigorous reporting to capture implementation learnings. A lack of evidence exists on how to improve equity, reach and engagement of lifestyle interventions that are non-stigmatising in underserved populations.

## Postpartum

### Significance of Weight and Lifestyle Postpartum

Postpartum weight retention (PPWR) carries significant risks to maternal and child health. An increase of three or more BMI units during 2 years between consecutive births poses a greater risk of adverse pregnancy outcomes in the subsequent pregnancy including preeclampsia, gestational diabetes, caesarean delivery, stillbirth, congenital anomalies, increased birth weight and LGA [70, 71]. In addition, PPWR 1 year after birth increases the risk of long-term maternal weight gain [72] and chronic disease risks in mothers [73]. The postpartum period is, therefore, a critical window for weight management in women to prevent long-term chronic disease risks and to prepare for subsequent healthy pregnancies.

### Challenges to Lifestyle Management Postpartum

Adverse lifestyle behaviours in the context of an obesogenic environment and psychosocial risk factors during the postpartum period contribute to PPWR [74]. Healthy dietary behaviours generally decline from pregnancy through to postpartum, marked by decreased fruit and vegetable intake and increased consumption of energy-dense foods [75–77]. Sleep deprivation after birth could also contribute to PPWR, with a positive association between short sleep duration and PPWR reported in a previous review [78]. Physical activity generally declines from late pregnancy through to the early postpartum period and increases gradually from 3 to 12 months postpartum although levels remain lower than that of pre-pregnancy [79–84]. Thus, poor diet, low physical activity and lack of sleep all contribute to PPWR. Mothers with certain sociodemographic characteristics including maternal age < 20 or > 40 years, primiparity, ethnic background (e.g. South Asia, Middle East and Africa compared to Western Europe backgrounds), unemployment, low income and low education may be at greater risk of PPWR [74, 85–88]. Psychological morbidities in the postpartum period such as stress and depression also contribute to PPWR [89, 90] although the relationship between anxiety and maternal obesity was less clear [91]. In addition to the numerous and indisputable health benefits to the child, breastfeeding also confers maternal health benefits which include a reduced risk of type 2 diabetes [92]. There is little evidence on the association between breastfeeding and PPWR [92].

Postpartum women face significant barriers to weight and lifestyle management which makes them more vulnerable and at higher risk in our increasingly obesogenic environment. Our recent systematic review summarized these barriers using the Capability, Opportunity, Motivation and Behaviour (COM-B) behaviour change framework [93]. In terms of capability, barriers reported included limited knowledge on how to safely resume exercise after birth, tiredness, lack of sleep and psychological morbidities such as stress and depression. In terms of opportunity, time constraints, prioritizing care for the child and household commitments over personal health, financial constraints and lack of suitable environment for exercise prevented healthy lifestyle in postpartum women. Barriers to social opportunities included lack of practical support from partners, lack of peer support for exercise and lack of lifestyle support from healthcare providers. Barriers to motivation included lack of self-confidence in exercise, unwillingness to change eating habits, lack of enjoyment of exercise or healthy food and low self-worth [93]. Interventions targeting postpartum women should address these barriers to improve engagement, which is the key challenge to implementation in this group [94]. We have previously demonstrated that addressing barriers to childcare, scheduling and accessibility led to an increase in engagement from 38 to 82% in a diabetes prevention program targeting postpartum who with a recent history of gestational diabetes [95].

### Intervention Studies Addressing Lifestyle Postpartum

Our systematic review and meta-analysis showed that interventions combining diet and physical activity are efficacious in reducing postpartum weight (33 studies, *n*=4960 women; mean difference (MD) −3.15 kg, 95% confidence interval, −4.34, −1.96) [96]. Lifestyle interventions for women postpartum have also been reported to significantly improve physical activity (standardized MD of 0.61, 95% CI (0.20 to 1.02)), but no significant effect on energy intake was reported [97–99], possibly due to limitations of dietary measurement methods preventing the detection of a small improvement in energy intake. While a combination of both diet and exercise produced the greatest weight loss, diet-only interventions are also efficacious for postpartum weight loss [100, 101] but not exercise-only interventions [100–103]. Despite the absence of significant weight loss, meta-analyses of exercise-only interventions reported improvements in maternal cardiovascular fitness [100] and postnatal depression [104]. In terms of intervention development, delivery by health professionals and a focus on behaviour change techniques on self-regulation such as goal-setting, problem-solving and self-monitoring could improve the effectiveness of postpartum lifestyle interventions [96, 97, 99].

### Gaps

Despite clear evidence on the importance and effectiveness of postpartum weight and lifestyle management, globally only 13% of maternal weight policies and guidelines address postpartum weight and lifestyle [105]. The UK National Institute for Health and Care Excellence (NICE) guidelines for Weight Management Before, During and After Pregnancy is one of the few that support the reduction of postpartum weight as a strategy for preventing maternal obesity [106]. Unlike the diabetes prevention programs which have progressed from efficacy trials to large-scale effectiveness trials in various settings [107, 108], there is a lack of similar real-world effectiveness studies in postpartum weight management. Instead, the existing interventions are at the level of small RCTs of moderate efficacy. There is a need for pragmatic trials and the development of effective models of care to implement the evidence on postpartum lifestyle and weight management.

## Health Economics

Lifestyle interventions across preconception, pregnancy and postpartum have clear evidence for their prevention of health conditions and events that carry a large cost to health service funders [109, 110]. For example, previous health economic analyses demonstrated a 40% higher health system cost with maternal obesity compared with pregnancies with a healthy maternal weight [111]. Strong evidence exists that lifestyle interventions in pregnancy are highly cost-effective, with diet interventions, and diet and physical activity interventions costing US$3,564 and US$1,465, respectively, per case of gestational diabetes, hypertensive disorders in pregnancy or caesarean birth avoided, respectively [69]. The same study also identified that physical activity interventions are likely to be cost-saving. Furthermore, studies demonstrating the cost-effectiveness of lifestyle interventions in pregnancy have not considered the longer-term health benefits [112] of prevention of caesarean delivery, gestational diabetes and obesity for mother and child. These interventions are thus likely to be even more cost-effective or cost-saving.

There is a gap in the literature on the cost-effectiveness of lifestyle interventions delivered preconception or postpartum. The cost-effectiveness of preconception lifestyle interventions has been noted as an area currently lacking evidence [113]. There is some evidence confirming that postpartum lifestyle interventions are highly cost-effective at between US$1,704 and US$ 7,889 per quality-adjusted life-year gained, which is well below traditional cost-effectiveness thresholds of US$50,000 [114]. This is an area warranting future investigation, as the long-term health benefits of both preconception and postpartum lifestyle interventions are likely to result in them being cost-saving or cost-effective to funders.

## Conclusions and Next Steps

With unhealthy weight gain and excess weight now highly prevalent among reproductive-age women, the adverse health impacts in the short and long-term for women and the next generation are undisputed and escalating. Helping women to achieve an optimal preconception weight and maintaining a healthy lifestyle is critical, given the adverse impacts of maternal obesity on fertility and pregnancy. Health messages should target women who are planning a pregnancy and those who are not, so that all women may achieve the best possible health and wellbeing, regardless of pregnancy intentions. During pregnancy, excessive GWG independently adversely impacts outcomes for mothers and babies. Simple healthy lifestyle interventions based on the strongest evidence successfully limit excess GWG and significantly improve outcomes for mothers and babies. These are proven to be cost-effective. Postpartum lifestyle and weight challenges continue with maternal and offspring implications into future pregnancies and later life, warranting weight management in this life stage. Healthy lifestyle education support is important for the health of women in the next generation, across the reproductive years, especially in pregnancy. Despite overwhelming evidence, there are significant implementation gaps in translating existing evidence into pragmatic models of care and real-world solutions in preconception, pregnancy and postpartum lifestyle and weight. Internationally, guidelines need to be updated to reflect the current evidence and implementation of healthy lifestyle support for women. Ultimately, international public health policies must contribute to the solution by making a healthy lifestyle the easier option to enable the prevention of unhealthy weight gain and reverse the significant adverse health outcomes for women and the next generation.
